# Systemic therapy for Asian patients with advanced BRAF V600‐mutant melanoma in a real‐world setting: A multi‐center retrospective study in Japan (B‐CHECK‐RWD study)

**DOI:** 10.1002/cam4.6438

**Published:** 2023-08-16

**Authors:** Kenjiro Namikawa, Takamichi Ito, Shusuke Yoshikawa, Koji Yoshino, Yukiko Kiniwa, Shuichi Ohe, Taiki Isei, Tatsuya Takenouchi, Hiroshi Kato, Satoru Mizuhashi, Satoshi Fukushima, Yosuke Yamamoto, Takashi Inozume, Yasuhiro Fujisawa, Osamu Yamasaki, Yasuhiro Nakamura, Jun Asai, Takeo Maekawa, Takeru Funakoshi, Shigeto Matsushita, Eiji Nakano, Kohei Oashi, Junji Kato, Hisashi Uhara, Takuya Miyagawa, Hiroshi Uchi, Naohito Hatta, Keita Tsutsui, Taku Maeda, Taisuke Matsuya, Hiroto Yanagisawa, Ikko Muto, Mao Okumura, Dai Ogata, Naoya Yamazaki

**Affiliations:** ^1^ Department of Dermatologic Oncology National Cancer Center Hospital Tokyo Japan; ^2^ Department of Dermatology, Graduate School of Medical Sciences Kyushu University Fukuoka Japan; ^3^ Department of Dermatology Shizuoka Cancer Center Shizuoka Japan; ^4^ Department of Dermatologic Oncology Tokyo Metropolitan Cancer and Infectious Diseases Center Komagome Hospital Tokyo Japan; ^5^ Department of Dermatology Shinshu University Matsumoto Japan; ^6^ Department of Dermatologic Oncology Osaka International Cancer Institute Osaka Japan; ^7^ Department of Dermatology Niigata Cancer Center Hospital Niigata Japan; ^8^ Department of Geriatric and Environmental Dermatology Nagoya City University Nagoya Japan; ^9^ Department of Dermatology and Plastic Surgery Kumamoto University Kumamoto Japan; ^10^ Department of Dermatology Chiba University Chiba Japan; ^11^ Department of Dermatology University of Tsukuba Tsukuba Japan; ^12^ Department of Dermatology Okayama University Graduate School of Medicine, Dentistry, and Pharmaceutical Sciences Okayama Japan; ^13^ Department of Skin Oncology/Dermatology Saitama Medical University International Medical Center Saitama Japan; ^14^ Department of Dermatology Kyoto Prefectural University of Medicine Kyoto Japan; ^15^ Department of Dermatology Jichi Medical University Hospital Tochigi Japan; ^16^ Department of Dermatology Keio University Tokyo Japan; ^17^ Department of Dermato‐Oncology/Dermatology National Hospital Organization Kagoshima Medical Center Kagoshima Japan; ^18^ Department of Dermatology Kobe University Kobe Japan; ^19^ Department of Dermatology Saitama Cancer Center Saitama Japan; ^20^ Department of Dermatology Sapporo Medical University Sapporo Japan; ^21^ Department of Dermatology University of Tokyo Tokyo Japan; ^22^ Department of Dermato‐Oncology National Hospital Organization Kyushu Cancer Center Fukuoka Japan; ^23^ Department of Dermatology Toyama Prefectural Central Hospital Toyama Japan; ^24^ Department of Dermatology Fukuoka University Fukuoka Japan; ^25^ Department of Plastic and Reconstructive Surgery Hokkaido University Sapporo Japan; ^26^ Department of Dermatology Asahikawa Medical University Asahikawa Japan; ^27^ Department of Dermatology Saitama Medical University Saitama Japan; ^28^ Department of Dermatology Kurume University Kurume Japan; ^29^ Present address: Department of Dermatologic Oncology The Cancer Institute Hospital of Japanese Foundation for Cancer Research Tokyo Japan; ^30^ Present address: Department of Dermatology Ehime University Ehime Japan; ^31^ Present address: Department of Dermatology Shimane University Faculty of Medicine Shimane Japan

**Keywords:** Asian, BRAF, immune‐checkpoint inhibitor, melanoma, targeted therapy

## Abstract

**Background:**

Anti‐PD‐1‐based immunotherapy is considered a preferred first‐line treatment for advanced BRAF V600‐mutant melanoma. However, a recent international multi‐center study suggested that the efficacy of immunotherapy is poorer in Asian patients in the non‐acral cutaneous subtype. We hypothesized that the optimal first‐line treatment for Asian patients may be different.

**Methods:**

We retrospectively collected data of Asian patients with advanced BRAF V600‐mutant melanoma treated with first‐line BRAF/MEK inhibitors (BRAF/MEKi), anti‐PD‐1 monotherapy (Anti‐PD‐1), and nivolumab plus ipilimumab (PD‐1/CTLA‐4) between 2016 and 2021 from 28 institutions in Japan.

**Results:**

We identified 336 patients treated with BRAF/MEKi (*n* = 236), Anti‐PD‐1 (*n* = 64) and PD‐1/CTLA‐4 (*n* = 36). The median follow‐up duration was 19.9 months for all patients and 28.6 months for the 184 pa tients who were alive at their last follow‐up. For patients treated with BRAF/MEKi, anti‐PD‐1, PD‐1/CTLA‐4, the median ages at baseline were 62, 62, and 53 years (*p* = 0.03); objective response rates were 69%, 27%, and 28% (*p* < 0.001); median progression‐free survival (PFS) was 14.7, 5.4, and 5.8 months (*p* = 0.003), and median overall survival (OS) was 34.6, 37.0 months, and not reached, respectively (*p* = 0.535). In multivariable analysis, hazard ratios (HRs) for PFS of Anti‐PD‐1 and PD‐1/CTLA‐4 compared with BRAF/MEKi were 2.30 (*p* < 0.001) and 1.38 (*p* = 0.147), and for OS, HRs were 1.37 (*p* = 0.111) and 0.56 (*p* = 0.075), respectively. In propensity‐score matching, BRAF/MEKi showed a tendency for longer PFS and equivalent OS with PD‐1/CTLA‐4 (HRs for PD‐1/CTLA‐4 were 1.78 [*p* = 0.149]) and 1.03 [*p* = 0.953], respectively). For patients who received second‐line treatment, BRAF/MEKi followed by PD‐1/CTLA‐4 showed poor survival outcomes.

**Conclusions:**

The superiority of PD‐1/CTLA‐4 over BRAF/MEKi appears modest in Asian patients. First‐line BRAF/MEKi remains feasible, but it is difficult to salvage at progression. Ethnicity should be considered when selecting systemic therapies until personalized biomarkers are available in daily practice. Further studies are needed to establish the optimal treatment sequence for Asian patients.

## Graphical abstract

This multi‐center real‐world data study including 336 Asian patients with advanced BRAF V600‐mutant melanoma showed that the superiority of Anti‐PD‐1/CTLA‐4 over BRAF/MEK inhibitor appears modest, and the BRAF/MEK inhibitor remains a feasible first‐line treatment option. Ethnicity should be considered when selecting systemic therapies until personalized biomarkers are available in daily clinical practice. Further studies are needed to establish the optimal treatment sequence for Asian patients.

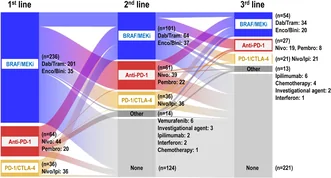

## 1 INTRODUCTION

Systemic treatment options for advanced BRAF V600‐mutant melanoma include BRAF/MEK inhibitors (BRAF/MEKi), anti‐PD‐1 monotherapy (Anti‐PD‐1), combination of anti‐PD‐1 antibody plus anti‐CTLA‐4 antibody (PD‐1/CTLA‐4), and recently added combination of relatlimab, anti‐LAG‐3 antibody, and nivolumab (relatlimab–nivolumab). In the past decade, a common clinical question was whether targeted therapies (TTs) or immune‐checkpoint inhibitors (ICIs) should be administered first.^1^, ^2^ Most retrospective studies and matching‐adjusted indirect comparisons,^3^, ^4^, ^5^, ^6^, ^7^, ^8^, ^9^, ^10^ but not all,^11^, ^12^, ^13^ have demonstrated favorable survival benefits for patients treated with ICI upfront. Recently, randomized controlled trials (RCTs) of the DREAMseq (*n* = 265)^14^ and SECOMBIT (*n* = 209)^15^ trials comparing treatment sequences of ICI and TT showed that upfront nivolumab plus ipilimumab (Nivo/Ipi) was associated with a higher 2‐year overall survival (OS) rate compared with upfront TT treatment arm. Therefore, anti‐PD‐1‐based immunotherapy is now considered a preferred first‐line treatment because of its potential for long‐term survival.^16^, ^17^

In Asian patients with advanced melanoma, the clinical efficacy of ICI, which is influenced by the interaction between tumor‐related factors and host immunity, is lower than that in Caucasian patients.^18^ The objective response rate (ORR) of first‐line nivolumab in the CheckMate 066 (nivolumab treatment arm, *n* = 210)^19^ and the ONO‐4538‐08 trial (*n* = 24),^20^, ^21^ which is a single‐arm phase II trial of first‐line nivolumab in Japan, was 43% and 35%, respectively. Similarly, the ORR of first‐line Nivo/Ipi in the CheckMate 067 (Nivo/Ipi treatment arm, *n* = 314)^22^ and the ONO‐4538‐17 trial (*n* = 30),^23^, ^24^ which is a single‐arm phase II trial of first‐line Nivo/Ipi in Japan, was 58% and 43%, respectively. One reason for the inferior clinical efficacy of ICI in Asian patients is the higher proportion of acral/mucosal subtypes, which have lower tumor mutational burden (TMB) than the non‐acral cutaneous subtype.^25^ The proportion of patients with acral/mucosal melanoma who participated in the ONO‐4538‐08 and ONO‐4538‐17 trials was 54% and 63%, respectively. Although it is unclear whether the difference in response to ICI between Caucasians and Asians is due to the difference in melanoma subtypes alone or due to ethnicity itself,^18^ a recent international multi‐center study suggested that ethnicity is also a possible reason. Clinical outcomes in East Asian, Hispanic, and African patients receiving Anti‐PD‐1 were poorer than that of Caucasian patients in the non‐acral cutaneous and unknown primary melanoma cohort.^26^ Differences in ultraviolet (UV)‐induced TMB due to differences in skin phototype between ethnicities may contribute to the varied outcome of Anti‐PD‐1 in non‐acral cutaneous melanoma. However, there was no clear difference in the efficacy of BRAF/MEKi between Caucasian and Asian patients; the ORR of the first‐line dabrafenib plus trametinib (Dab/Tram) of the COMBI‐d/COMBI‐v (*n* = 563)^27^ and Japanese single‐arm phase I/II trial of Dab/Tram (*n* = 12)^28^ was 68% and 67%, respectively.

Based on these findings, we hypothesized that the optimal first‐line treatment for Asian patients may be different from that for Caucasian patients. This study aimed to assess the efficacy of first‐line BRAF/MEKi, Anti‐PD‐1, and PD‐1/CTLA‐4 in Asian patients with advanced BRAF V600‐mutant melanoma in a real‐world setting.

## 2 MATERIALS AND METHODS

### 2.1 Patients

Patients aged ≥20 years diagnosed with unresectable/metastatic stage III/IV BRAF V600‐mutant melanoma and treated with first‐line BRAF/MEKi, Anti‐PD‐1, or PD‐1/CTLA‐4 from April 2016 to March 2021 were included. This study was conducted in compliance with the Ethical Guidelines for Medical and Biological Research Involving Human Subjects,^29^ and the Declaration of Helsinki. Permission to perform the present study and a waiver for informed consent were obtained from the National Cancer Center Research Ethics Review Committee (IRB No. 2021‐238). Permission to perform the present study was obtained at each institution as well.

### 2.2 Study design and data collection

In this multi‐center retrospective study across 28 institutions in Japan, patient demographics (age, sex, and ethnicity), disease characteristics (melanoma subtype, genotype of BRAF mutation, and PD‐L1 expression), baseline characteristics (Eastern Cooperative Oncology Group performance status [PS], pathological stage based on the 8th edition of the American Joint Committee of Cancer [AJCC] staging system for cutaneous melanoma, serum lactate dehydrogenase [LDH], number of metastatic organ sites, and prior history of adjuvant therapy) at the time of first‐line treatment initiation, efficacy and safety of first‐line to third‐line treatment, and survival status at the time of last follow‐up, were collected from medical record at each institution.

### 2.3 Efficacy and safety assessments

Clinical outcomes included ORR, disease control rate (DCR), progression‐free survival (PFS), PFS2, and OS. Tumor response was assessed at each institution based on the Response Evaluation Criteria in Solid Tumors, version 1.1. ORR was defined as the proportion of patients with partial response (PR) or complete response (CR). DCR was defined as the proportion of patients with stable disease (SD), PR, or CR. PFS was defined as the time from the start of first‐line treatment to disease progression, death, or last follow‐up. In patients who received second‐line treatment, PFS2, defined as the time from the first‐line treatment start to progression after the second‐line treatment start, death, or last follow‐up, was assessed. OS was defined as the time from the start of the first‐line treatment to death or last follow‐up. The severity of the adverse event (AE) was graded according to the National Cancer Institute Common Terminology Criteria for Adverse Events, version 4.0. Only grade ≥3 AEs or any grade AEs leading to treatment discontinuation was collected because lower‐grade AEs are likely to be underreported in retrospective studies.

### 2.4 Statistical analyses

Baseline characteristics were compared using the chi‐square and Kruskal–Wallis tests for categorical and continuous variables, respectively. Differences in ORR and DCR between the groups were tested using Fisher's exact test. The Clopper–Pearson exact method was used to calculate the 95% confidence interval (CI) of the ORR and DCR. The Kaplan–Meier method was used to estimate PFS, PFS2, and OS, and differences between groups were assessed using the log‐rank test. The Cox proportional hazard regression model was used to calculate hazard ratios (HRs). As the statistical software used in this study did not support propensity‐score matching between multiple groups, each pair of first‐line treatment groups was evaluated. Covariates were chosen based on clinical practice and well‐known prognostic factors, and multivariable logistic regression analysis was used to estimate propensity scores. Caliper was initially set at 0.2 times the standard deviation of the propensity score. To assess the quality of matching, standardized differences for all covariates both in the original and matched cohorts were calculated. The caliper was lowered until standardized differences for all covariates in the matched cohort were <0.1. Statistical significance was set at *p* < 0.05. All statistical analyses were performed using SPSS Statistics software, version 23.0 (IBM Corporation).

## 3 RESULTS

### 3.1 Patient characteristics

**FIGURE 1:**
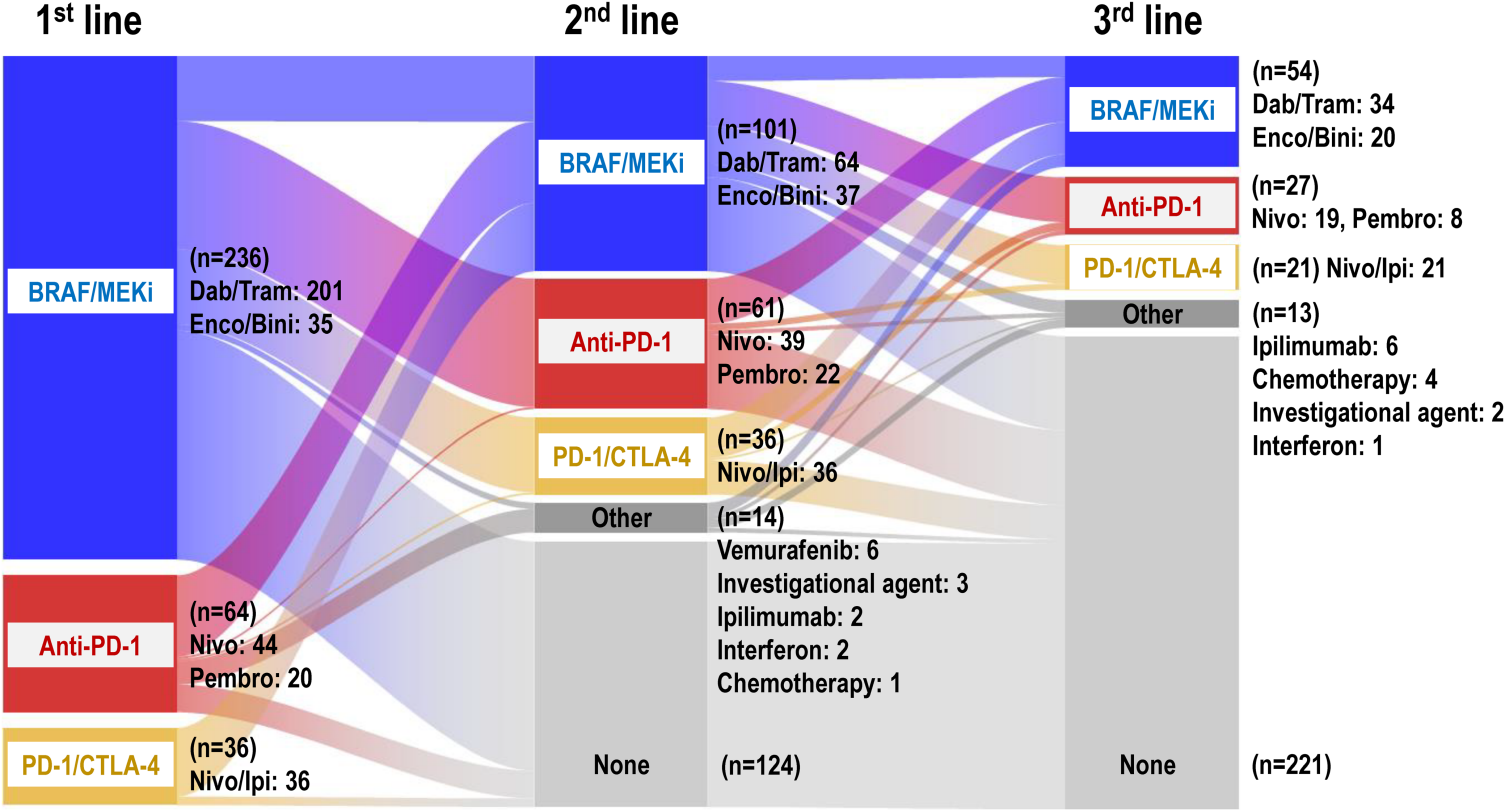
Diagram of the treatment sequence and patient disposition from first‐ to third‐line treatment. BRAF/MEKi, BRAF plus MEK inhibitors; Anti‐PD‐1, anti‐PD‐1 antibody; PD‐1/CTLA‐4, anti‐PD‐1 antibody plus anti‐CTLA‐4 antibody; Dab/Tram, dabrafenib plus trametinib; Enco/Bini, encorafenib plus binimetinib; Nivo, nivolumab; Pembro, pembrolizumab; NIvo/Ipi, nivolumab plus ipilimumab.

After excluding 74 patients who did not meet the inclusion criteria (Figure S1), 336 Asian patients with a median age of 61 years (range, 20–91) were included in this study. The patients were treated with first‐line BRAF/MEKi (*n* = 236, 70%), anti‐PD‐1 (*n* = 64, 19%), or PD‐1/CTLA‐4 (*n* = 36, 11%) (Table 1; Figure 1). The median follow‐up duration was 19.9 months (range, 0.1–94.7) for all patients, and 28.6 months (range, 1.7–94.7) for the 184 patients who were alive at their last follow‐up. Although acral and mucosal subtypes are common in an Asian population, these subtypes accounted for 6.5% and 3.0%, respectively, because of the lower positivity rate of BRAF mutation in these subtypes. The BRAF genotype was not specified in 36 (11%) patients whose tumors were examined using the Cobas® 4800 BRAF V600 Mutation Test, which cannot distinguish between V600E and V600K. Median age, BRAF genotype, and AJCC stage varied significantly between the first‐line treatment groups. There was a trend toward a higher percentage of PD‐L1 expression positivity in the PD‐1/CTLA‐4 treatment group, but PD‐L1 expression was not examined in more than half of the entire cohort (Table 1).

**TABLE 1:** Baseline characteristics of the patients in the entire cohort and sub‐cohort stratified by first‐line treatment.

		First‐line treatment			
Characteristic	Total (*n* = 336)	BRAF/MEKi (*n* = 236)	Anti‐PD‐1 (*n* = 64)	PD‐1/CTLA‐4 (*n* = 36)	*p*‐Value
Age (years)					
Median (range)	61 (20–91)	62 (20–91)	62 (26–86)	53 (22–85)	0.03
Sex					
Female	166 (49.4)	119 (50.4)	28 (43.8)	19 (52.8)	0.58
Male	170 (50.6)	117 (49.6)	36 (56.3)	17 (47.2)	
ECOG PS					
0	274 (81.5)	189 (80.0)	57 (89.1)	28 (77.8)	0.59
1	40 (11.9)	30 (12.7)	6 (9.4)	4 (11.1)	
2	10 (3.0)	8 (3.4)	1 (1.6)	1 (2.8)	
3	9 (2.7)	7 (3.0)	0 (0.0)	2 (5.6)	
4	3 (0.9)	2 (0.8)	0 (0.0)	1 (2.8)	
Subtype					
Non‐acral cutaneous	279 (83.0)	199 (84.3)	54 (84.4)	26 (72.2)	0.17
Acral	22 (6.5)	16 (6.8)	4 (6.3)	2 (5.6)	
Mucosal	10 (3.0)	7 (3.0)	0 (0.0)	3 (8.3)	
Unknown primary	25 (7.4)	14 (5.9)	6 (9.4)	5 (13.9)	
BRAF mutation					
V600E	266 (79.2)	194 (82.2)	45 (70.3)	27 (75.0)	0.01
V600K	33 (9.8)	23 (9.7)	6 (9.4)	4 (11.1)	
V600R	1 (0.3)	0 (0.0)	0 (0.0)	1 (2.8)	
V600 (unspecified)	36 (10.7)	19 (8.1)	13 (20.3)	4 (11.1)	
Stage (AJCC 8th edition)					
III (unresectable)	71 (21.1)	53 (22.5)	12 (18.8)	6 (16.7)	0.01
IV (M1a)	65 (19.3)	42 (17.8)	17 (26.6)	6 (16.7)	
IV (M1b)	74 (22.0)	46 (19.5)	21 (32.8)	7 (19.4)	
IV (M1c)	89 (26.5)	69 (29.2)	12 (18.8)	8 (22.2)	
IV (M1d)	37 (11.0)	26 (11.0)	2 (3.1)	9 (25.0)	
Metastatic organ sites					
1	180 (53.6)	127 (53.8)	40 (62.5)	13 (36.1)	0.13
2	78 (23.2)	53 (22.5)	14 (21.9)	11 (30.6)	
≥3	78 (23.2)	56 (23.7)	10 (15.6)	12 (33.3)	
LDH					
Normal	239 (71.1)	166 (70.3)	49 (76.6)	24 (66.7)	0.84
Elevated (<2 × ULN)	70 (20.8)	50 (21.2)	11 (17.2)	9 (25.0)	
Elevated (≥2 × ULN)	27 (8.0)	20 (8.5)	4 (6.3)	3 (8.3)	
PD‐L1					
<1%	54 (16.1)	39 (16.5)	10 (15.6)	5 (13.9)	0.06
≥1%	74 (22.0)	46 (19.5)	13 (20.3)	15 (41.7)	
Not performed	208 (61.9)	151 (64.0)	41 (64.1)	16 (44.4)	
Adjuvant therapy					
None	215 (64.0)	154 (65.3)	38 (59.4)	23 (63.9)	0.72
Dab/Tram	19 (5.7)	12 (5.1)	4 (6.3)	3 (8.3)	
Anti‐PD‐1	14 (4.2)	11 (4.7)	1 (1.6)	2 (5.6)	
IFNs/Chemo	88 (26.2)	59 (25.0)	21 (32.8)	8 (22.2)	

Abbreviations: AJCC, American Joint Committee on Cancer; Anti‐PD‐1, anti‐PD‐1 antibody; BRAF/MEKi, BRAF plus MEK inhibitors; Chemo, chemotherapy; Dab/Tram, dabrafenib plus trametinib; ECOG PS, Eastern Cooperative Oncology Group performance status; IFNs, interferons; LDH, lactate dehydrogenase; PD‐1/CTLA‐4, anti‐PD‐1 antibody plus anti‐CTLA‐4 antibody; ULN, upper limit of normal.

### 3.2 Survival outcomes of the entire cohort

**FIGURE 2:**
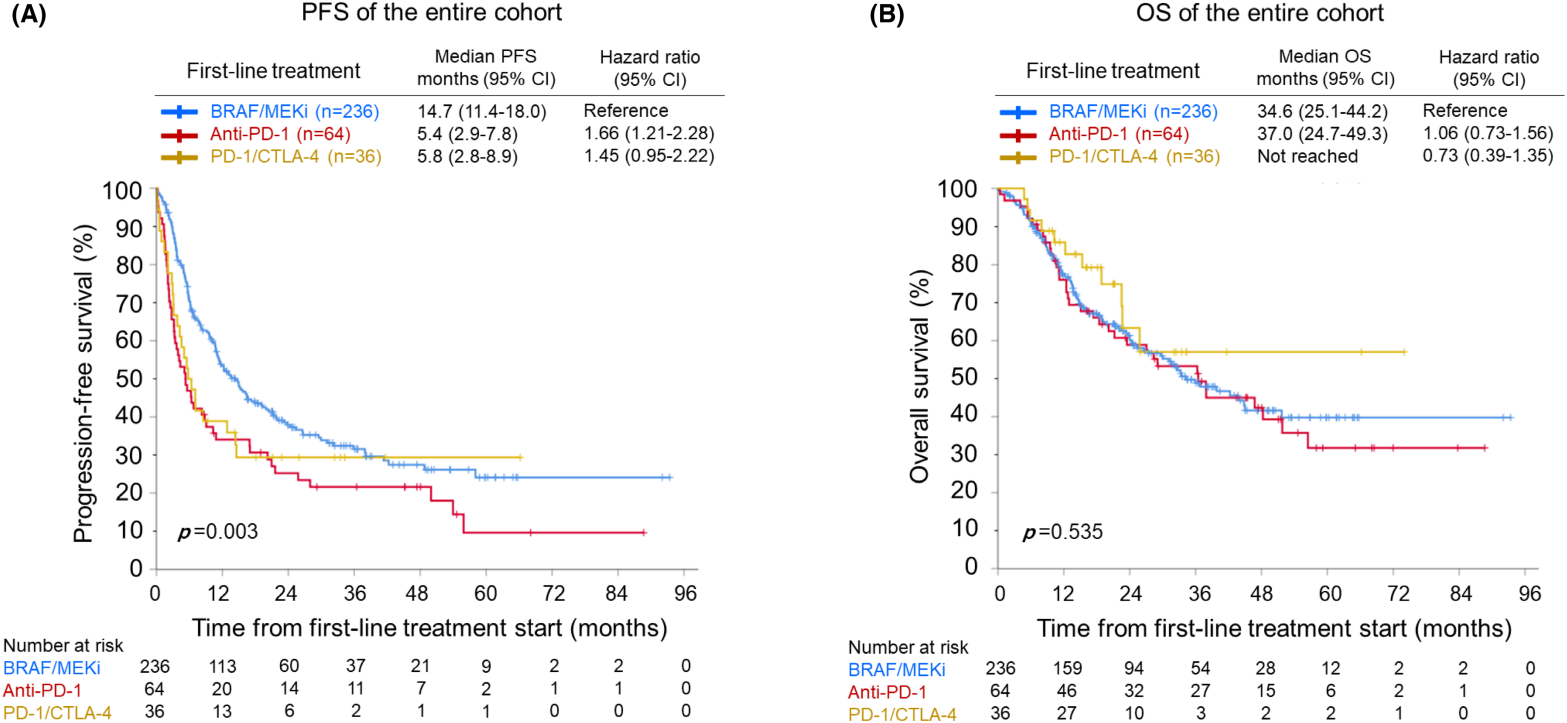
Kaplan–Meier curve of the entire cohort for progression‐free survival (PFS) (A) and overall survival (OS) (B) according to first‐line treatment of BRAF plus MEK inhibitors (BRAF/MEKi), anti‐PD‐1 antibody (Anti‐PD‐1), and anti‐PD‐1 antibody plus anti‐CTLA‐4 antibody (PD‐1/CTLA‐4).

The median PFS (95% CI) of the first‐line BRAF/MEKi, Anti‐PD‐1, and PD‐1/CTLA‐4 treatment arms were 14.7 (11.4–18.0), 5.4 (2.9–7.8), and 5.8 (2.8–8.9) months, respectively (*p* = 0.003) (Figure 2A). The 2‐year PFS rates of the first‐line treatment arms were 37.9%, 25.2%, and 29.4%, respectively. In multivariable analysis, HRs for PFS of Anti‐PD‐1 and PD‐1/CTLA‐4 compared with BRAF/MEKi were 2.30 (1.65–3.21, *p* < 0.001) and 1.38 (0.89–2.12, *p* = 0.147), respectively. Other variables significantly associated with poor PFS were PS 2–4, stage IV (M1c/M1d), and elevated LDH (Table 2).

**TABLE 2:** Univariate and multivariable analysis for progression‐free survival.

		Univariate (PFS)		Multivariable (PFS)	
Variables	*n*	HR (95% CI)	*p*‐Value	HR (95% CI)	*p*‐Value
First‐line treatment					
BRAF/MEKi	236	Ref		Ref	
Anti‐PD‐1	64	1.66 (1.21–2.28)	0.002	2.30 (1.65–3.21)	<0.001
PD‐1/CTLA‐4	36	1.45 (0.95–2.22)	0.085	1.38 (0.89–2.12)	0.147
Age					
<65 years	194	Ref		Ref	
≥65 years	142	0.94 (0.73–1.23)	0.668	0.88 (0.67–1.15)	0.340
Sex					
Female	166	Ref		Ref	
Male	170	1.02 (0.79–1.33)	0.871	1.06 (0.81–1.38)	0.666
ECOG PS					
0–1	314	Ref		Ref	
2–4	22	2.64 (1.68–4.15)	<0.001	2.49 (1.54–4.03)	<0.001
Subtype					
NAC/UP	304	Ref		Ref	
Acral/mucosal	32	0.73 (0.45–1.16)	0.182	1.11 (0.69–1.79)	0.678
BRAF mutation					
V600E	266	Ref		Ref	
V600K/R/unspecified	70	1.10 (0.80–1.51)	0.555	1.35 (0.97–1.89)	0.076
Stage (AJCC 8th edition)					
III/IV (M1a/M1b)	210	Ref		Ref	
IV (M1c/M1d)	126	2.13 (1.64–2.79)	<0.001	2.05 (1.42–2.97)	<0.001
Metastatic organ sites					
1–2	258	Ref		Ref	
≥3	78	2.06 (1.54–2.77)	<0.001	1.09 (0.74–1.60)	0.680
LDH					
Normal	239	Ref		Ref	
Elevated	97	2.26 (1.71–2.98)	<0.001	2.01 (1.49–2.73)	<0.001

Abbreviations: AJCC, American Joint Committee on Cancer; Anti‐PD‐1, anti‐PD‐1 antibody; BRAF/MEKi, BRAF plus MEK inhibitors; CI, confidence interval; ECOG PS, Eastern Cooperative Oncology Group performance status; HR, hazard ratio; LDH, lactate dehydrogenase; N, number of patients; NAC, non‐acral cutaneous; PD‐1/CTLA‐4, anti‐PD‐1 antibody plus anti‐CTLA‐4 antibody; PFS, progression‐free survival; Ref, reference; UP, unknown primary.

The median OS (95% CI) of first‐line BRAF/MEKi, Anti‐PD‐1, and PD‐1/CTLA‐4 treatment arms were 34.6 (25.1–44.2) and 37.0 (24.7–49.3) months and not reached, respectively (*p* = 0.535) (Figure 2B). The 2‐year OS rates of the first‐line treatment arms were 60.7%, 58.9%, and 63.4%, respectively. In multivariable analysis, HRs for OS of Anti‐PD‐1 and PD‐1/CTLA‐4 compared with BRAF/MEKi were 1.37 (0.93–2.03, *p* = 0.111) and 0.56 (0.30–1.06, *p* = 0.075), respectively. Other variables significantly associated with poor OS were PS 2–4, stage IV (M1c/M1d), and elevated LDH (Table 3).

**TABLE 3:** Univariate and multivariable analysis for overall survival.

		Univariate (OS)		Multivariable (OS)	
Variables	*n*	HR (95% CI)	*p*‐Value	HR (95% CI)	*p*‐respect value
First‐line treatment					
BRAF/MEKi	236	Ref		Ref	
Anti‐PD‐1	64	1.06 (0.73–1.56)	0.748	1.37 (0.93–2.03)	0.111
PD‐1/CTLA‐4	36	0.73 (0.39–1.35)	0.315	0.56 (0.30–1.06)	0.075
Age					
<65 years	194	Ref		Ref	
≥65 years	142	1.23 (0.90–1.70)	0.198	1.23 (0.88–1.72)	0.229
Sex					
Female	166	Ref		Ref	
Male	170	1.13 (0.82–1.55)	0.462	1.12 (0.81–1.54)	0.505
ECOG PS					
0–1	314	Ref		Ref	
2–4	22	4.45 (2.70–7.35)	<0.001	3.57 (2.08–6.11)	<0.001
Subtype					
NAC/UP	304	Ref		Ref	
Acral/Mucosal	32	0.62 (0.34–1.14)	0.125	1.12 (0.60–2.09)	0.724
BRAF mutation					
V600E	266	Ref		Ref	
V600K/R/unspecified	70	1.03 (0.70–1.51)	0.895	1.33 (0.88–2.00)	0.177
Stage (AJCC 8th edition)					
III/IV (M1a/M1b)	210	Ref		Ref	
IV (M1c/M1d)	126	3.01 (2.18–4.16)	<0.001	2.66 (1.79–3.96)	<0.001
Metastatic organ sites					
1–2	258	Ref		Ref	
≥3	78	2.42 (1.71–3.42)	<0.001	0.996 (0.63–1.57)	0.988
LDH					
Normal	239	Ref		Ref	
Elevated	97	2.98 (2.14–4.13)	<0.001	2.39 (1.66–3.44)	<0.001

Abbreviations: AJCC, American Joint Committee on Cancer; Anti‐PD‐1, anti‐PD‐1 antibody; BRAF/MEKi, BRAF plus MEK inhibitors; CI, confidence interval; ECOG PS, Eastern Cooperative Oncology Group performance status; HR, hazard ratio; LDH, lactate dehydrogenase; N, number of patients; NAC, non‐acral cutaneous; OS, overall survival; PD‐1/CTLA‐4, anti‐PD‐1 antibody plus anti‐CTLA‐4 antibody; Ref, reference; UP, unknown primary.

### 3.3 Survival outcomes of the matched cohort

**FIGURE 3:**
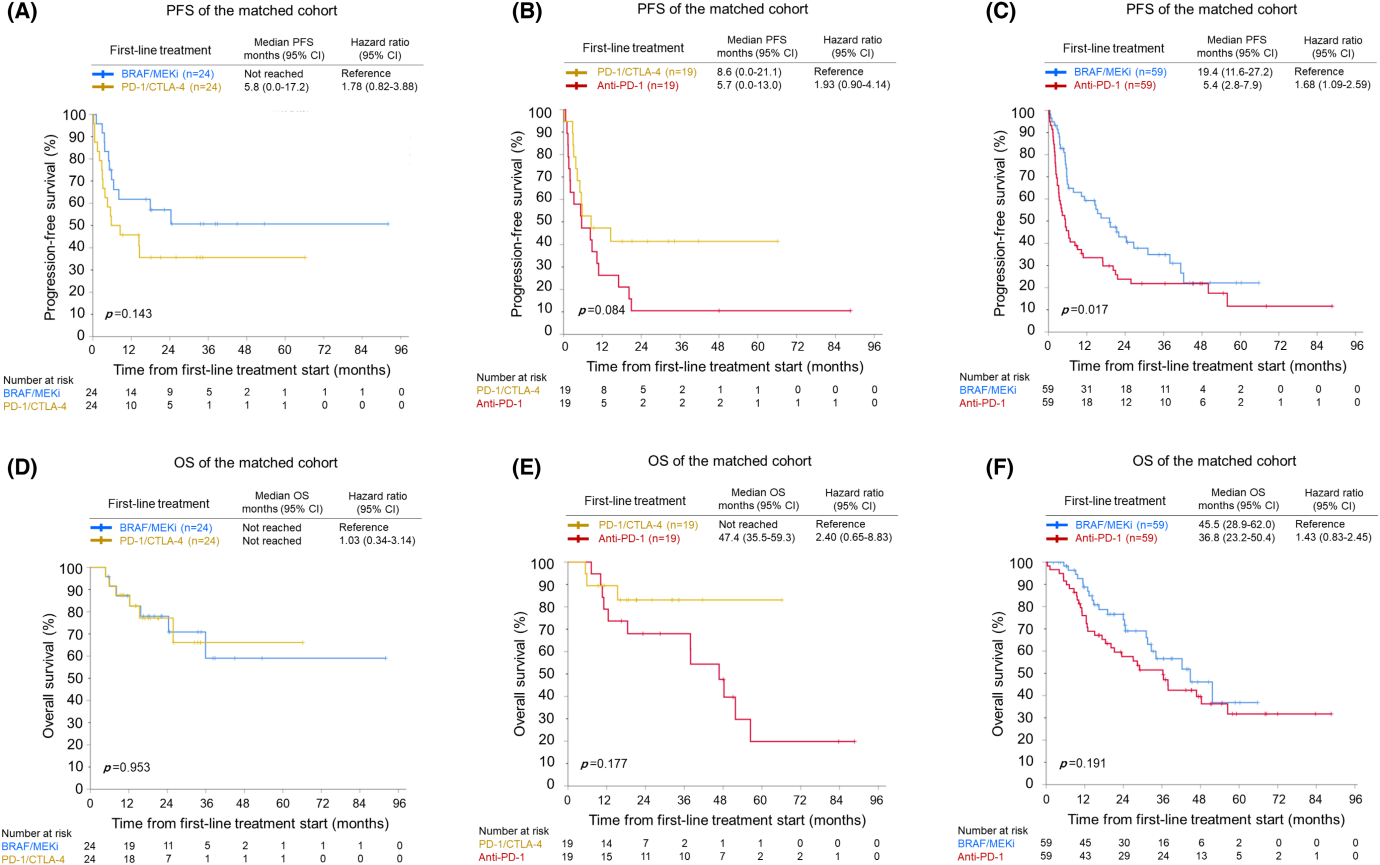
Kaplan–Meier curve of the matched cohort for progression‐free survival (PFS) (A)–(C) and overall survival (OS) (D)–(F) according to first‐line treatment of BRAF plus MEK inhibitors (BRAF/MEKi) versus anti‐PD‐1 antibody plus anti‐CTLA‐4 antibody (PD‐1/CTLA‐4) (A) and (D), PD‐1/CTLA‐4 versus anti‐PD‐1 antibody (Anti‐PD‐1) (B) and (E), and BRAF/MEKi versus Anti‐PD‐1 (C) and (F).

Three matched pairs of each of the two first‐line treatment groups were identified by propensity‐score matching: BRAF/MEKi (*n* = 24) versus PD‐1/CTLA‐4 (*n* = 24), PD‐1/CTLA‐4 (*n* = 19) versus anti‐PD‐1 (*n* = 19), and BRAF/MEKi (*n* = 59) versus Anti‐PD‐1 (*n* = 59) (Table S1A–C; Figure 3). The calipers were finally set at 0.0004, 0.0002, and 0.00001, respectively. In the comparison between BRAF/MEKi and PD‐1/CTLA‐4, BRAF/MEKi showed a tendency for longer PFS (HR for PD‐1/CTLA‐4, 1.78 [0.82–3.88], *p* = 0.149) and equivalent OS (1.03 [0.34–3.14], *p* = 0.953). In the comparison between PD‐1/CTLA‐4 and Anti‐PD‐1, PD‐1/CTLA‐4 showed a tendency for longer PFS (HR for Anti‐PD‐1, 1.93 [0.90–4.14], *p* = 0.089) and a tendency for longer OS (2.40 [0.65–8.83], *p* = 0.189). In the comparison between BRAF/MEKi and Anti‐PD‐1, BRAF/MEKi showed significantly longer PFS (HR for Anti‐PD‐1, 1.68 [1.09–2.59], *p* = 0.018) and a tendency for longer OS (1.43 [0.83–2.45], *p* = 0.194).

### 3.4 Subgroup analysis

Subgroup analysis for PFS and OS was performed according to PS (0–1/≥2), melanoma subtype (non‐acral cutaneous or unknown primary/acral or mucosal), BRAF genotype (V600E/V600K, R, or unspecified), AJCC stage (III or IV [M1a, M1b]/IV [M1c, M1d]), number of metastatic organ sites (1–2/≥3), and serum LDH at baseline (normal/elevated) (Figure S2). In terms of PFS, BRAF/MEKi demonstrated significantly favorable outcomes in the PS ≥2, stage IV (M1b/M1d), and elevated LDH subgroups, while PD‐1/CTLA‐4 demonstrated significantly favorable outcomes in the BRAF V600K/R/unspecified subgroup. In terms of OS, although no significant difference between first‐line treatment groups was observed in any subgroups, BRAF/MEKi showed favorable outcomes in the elevated LDH subgroup, while PD‐1/CTLA‐4 showed favorable outcomes in PS 0–1, BRAF V600K/R/unspecified, stage III/IV (M1a/M1b), metastatic organ sites ≤2, and normal LDH subgroups.

### 3.5 Response and survival outcome according to treatment sequence

The ORR of first‐line BRAF/MEKi, Anti‐PD‐1, and PD‐1/CTLA‐4 were 69%, 27%, and 28%, respectively (*p* < 0.001). The superiority of BRAF/MEKi in ORR was maintained in the second‐line setting (64%, 15%, and 17%, respectively) (*p* < 0.001), but disappeared in the third‐line setting (32%, 15%, and 24%, respectively) (*p* = 0.251) (Table S2).

In patients who received second‐line treatment, four treatment sequences, BRAF/MEKi followed by Anti‐PD1 (*n* = 60), BRAF/MEKi followed by PD‐1/CTLA‐4 (*n* = 35), Anti‐PD‐1 followed by BRAF/MEKi (*n* = 38), and PD‐1/CTLA‐4 followed by BRAF/MEKi (*n* = 32), were assessed (Figure S3). In both PFS2 and OS, PD‐1/CTLA‐4 followed by BRAF/MEKi showed the most favorable survival outcomes, while BRAF/MEKi followed by PD‐1/CTLA‐4 showed the poorest survival outcomes.

### 3.6 Adverse events

The proportion of patients who experienced grade ≥3 AEs or any grade AEs leading to treatment discontinuation in the first‐line BRAF/MEKi, Anti‐PD‐1, and PD‐1/CTLA‐4 treatment groups were 35%, 22%, and 75%, respectively (Table S3). The most frequent AE in each first‐line treatment group were pyrexia (*n* = 32, 14%), pneumonitis (*n* = 3, 5%), and hepatitis (*n* = 7, 20%), respectively.

## 4 DISCUSSION

In this multi‐center real‐world data study, we analyzed the clinical efficacy of first‐line BRAF/MEKi, Anti‐PD‐1, and PD‐1/CTLA‐4 therapies in 336 Asian patients with advanced BRAF V600‐mutant melanoma. We found that PD‐1/CTLA‐4 showed a slightly longer OS with higher toxicity than other treatment options, however, its superiority over BRAF/MEKi appears modest in Asian patients. To the best of our knowledge, this is the first real‐world data study including more than 300 Asian patients with advanced BRAF V600‐mutant melanoma. Previous studies, with a few exceptions,^3^, ^11^ did not include both Anti‐PD‐1 and PD‐1/CTLA‐4, or combined them together as ICIs. Since BRAF V600‐mutant melanoma responded better to PD‐1/CTLA‐4 in the CheckMate 067 trial, Anti‐PD‐1 and PD‐1/CTLA‐4 should be evaluated separately. In phase III RCTs, only PD‐1/CTLA‐4 was selected as the ICI,^14^, ^15^ however, considering the higher frequency of immune‐related AEs, there should be a role for Anti‐PD‐1 in clinical practice.

For first‐line treatment, we concluded that BRAF/MEKi is consistently efficacious in terms of PFS, based on the results of both multivariable and propensity‐score matching analyses. For OS, PD‐1/CTLA‐4 showed longer survival in the multivariable analysis; however, its superiority over BRAF/MEKi was not shown in the propensity‐score matching analysis. The 2‐year OS of PD‐1/CTLA‐4 in the present study was 63.4%, which is lower than that observed in the DREAMseq and SECOMBIT trials (71.8% and 73%, respectively). In contrast, the 2‐year OS of BRAF/MEKi in the present study was 60.7%, which is comparable to that observed in the DREAMseq and SECOMBIT trials (51.5% and 65%, respectively). Therefore, it appears that the efficacy of ICIs in Asians is lower than in Caucasians, whereas the efficacy of TTs is equivalent. Therefore, PD‐1/CTLA‐4 may not always be the best choice of first‐line treatment for Asian patients. According to the results of the subgroup analysis, patients with PS ≤1, V600K/R/unspecified genotype, stage III/IV (M1a/M1b), metastatic organ sites ≤2, or normal LDH responded better to PD‐1/CTLA‐4, and patients with elevated LDH responded better to BRAF/MEKi. V600K genotypes appear to be less reliant on ERK pathway activation, and thus, may benefit less from BRAF/MEKi. V600K genotypes are observed more frequently in older adults and in those with chronic sun‐damaged skin; therefore, these populations are likely to have a high TMB, which is associated with a higher response to immunotherapy.^2^, ^30^ Elevated LDH represents not only a higher tumor burden but also tumor‐derived immune suppression,^31^, ^32^ therefore, patients with elevated LDH are less likely to respond to Anti‐PD‐1. Anti‐PD‐1 was inferior to PD‐1/CTLA‐4 both in terms of PFS and OS, which is consistent with the BRAF‐mutant cohort of the CheckMate 067 trial. Although the reasons why BRAF‐mutant melanoma responds better to combination ICIs remain unclear, differences in the tumor microenvironment (TME) between BRAF‐mutant and wild‐type melanoma are indicated. A hypothetical model suggested that the BRAF V600‐mutation can upregulate CD73 expression resulting in the increase of adenosine, which can lead to immunosuppressive TME by activating regulatory T‐cells.^33^, ^34^ Another study demonstrated that the TME of BRAF‐mutant melanoma is associated with decreased CD8^+^ T‐cells and increased B‐cells and CD4^+^ T‐cells, which is distinct from BRAF wild‐type.^35^ Compared with BRAF/MEKi, Anti‐PD‐1 was also inferior both in terms of PFS and OS. In clinical practice, Anti‐PD‐1 is more easily selected than PD‐1/CTLA‐4 because salvage treatment with BRAF/MEKi is available. However, our results suggested that combination immunotherapy should be offered for patients who can tolerate it. Instead of Anti‐PD‐1, relatlimab–nivolumab is expected to be used more widely in the near future. However, it remains unclear whether relatlimab–nivolumab can replace PD‐1/CTLA‐4 because long‐term data is currently insufficient.

As for the treatment sequence, TT responded well both in first‐ and second‐line settings. In another study, BRAF/MEKi showed meaningful activity in patients who developed resistance to anti‐PD‐1‐based immunotherapy,^36^ and it was efficacious across all lines of therapy.^37^ On the other hand, responses to second‐line ICI therapy were not as good as those to first‐line ICI therapy. This is consistent with recent research; tumor relapse after TT is cross‐resistant to ICI,^38^ whereas treatment with ICI followed by TT leads to a durable response.^39^ In Asian patients, the risk–benefit balance of PD‐1/CTLA‐4 seems to be poor because its efficacy is worse, while immune‐related AEs occur with similar frequency. Therefore, first‐line BRAF/MEKi remains feasible, but it may be difficult to salvage with ICI during progression.

A possible reason for the lower efficacy of ICI in Asian patients with BRAF V600‐mutant melanoma is the lower UV‐induced TMB due to skin phototype, which is around type III–IV in Japanese populations according to Fitzpatrick skin phototyping. Although there is no actual TMB data, a multi‐center study in France showed that the location of primary melanoma according to sun exposure is associated with TMB.^40^ Considering the positive correlation between primary melanoma location and TMB, it is not surprising that skin phototype, which is associated with UV protection, also correlates with TMB. Therefore, we suggest that ethnicity should be taken into account as globalization increases when selecting systemic therapies until personalized biomarkers, including TMB and UV signature, are available in daily clinical practice. However, further studies are needed to confirm our hypothesis because other ethnic differences, such as HLA^41^, ^42^ or the microbiome,^43^, ^44^ also can contribute to the varied efficacy of ICI among ethnic groups.

The limitations of this study included a small sample size and confounding factors due to the retrospective nature of the study. However, an effort was made to minimize these limitations by performing multivariable analysis and propensity‐score matching.

## 5 CONCLUSIONS

In Asian patients with advanced BRAF V600‐mutant melanoma, the superiority of first‐line PD‐1/CTLA‐4 over BRAF/MEKi appears modest. BRAF/MEKi remains a feasible first‐line treatment option; however, it may be difficult to salvage with ICI during disease progression. Our findings suggest that ethnicity should be considered when selecting systemic therapies until personalized biomarkers are available in daily clinical practice. Further studies including clinical trials are needed to establish the optimal treatment sequence for Asian patients.

## AUTHOR CONTRIBUTIONS

**Kenjiro Namikawa:** Conceptualization (lead); data curation (lead); formal analysis (lead); funding acquisition (lead); investigation (lead); methodology (lead); project administration (lead); resources (lead); software (lead); supervision (lead); validation (lead); visualization (lead); writing – original draft (lead). **Takamichi Ito:** Conceptualization (equal); data curation (equal); investigation (equal); methodology (equal); resources (equal); validation (equal); visualization (equal); writing – review and editing (equal). **Shusuke Yoshikawa:** Conceptualization (equal); data curation (equal); investigation (equal); methodology (equal); resources (equal); validation (equal); visualization (equal); writing – review and editing (equal). **Koji Yoshino:** Conceptualization (equal); data curation (equal); investigation (equal); methodology (equal); resources (equal); validation (equal); visualization (equal); writing – review and editing (equal). **Yukiko Kiniwa:** Conceptualization (equal); data curation (equal); investigation (equal); methodology (equal); resources (equal); validation (equal); visualization (equal); writing – review and editing (equal). **Shuichi Ohe:** Conceptualization (equal); data curation (equal); investigation (equal); methodology (equal); resources (equal); validation (equal); visualization (equal); writing – review and editing (equal). **Taiki Isei:** Conceptualization (equal); data curation (equal); investigation (equal); methodology (equal); resources (equal); validation (equal); visualization (equal); writing – review and editing (equal). **Tatsuya Takenouchi:** Conceptualization (equal); data curation (equal); investigation (equal); methodology (equal); resources (equal); validation (equal); visualization (equal); writing – review and editing (equal). **Hiroshi Kato:** Conceptualization (equal); data curation (equal); investigation (equal); methodology (equal); resources (equal); validation (equal); visualization (equal); writing – review and editing (equal). **Satoru Mizuhashi:** Conceptualization (equal); data curation (equal); investigation (equal); methodology (equal); resources (equal); validation (equal); visualization (equal); writing – review and editing (equal). **Satoshi Fukushima:** Conceptualization (equal); data curation (equal); investigation (equal); methodology (equal); resources (equal); validation (equal); visualization (equal); writing – review and editing (equal). **Yosuke Yamamoto:** Conceptualization (equal); data curation (equal); investigation (equal); methodology (equal); resources (equal); validation (equal); visualization (equal); writing – review and editing (equal). **Takashi Inozume:** Conceptualization (equal); data curation (equal); investigation (equal); methodology (equal); resources (equal); validation (equal); visualization (equal); writing – review and editing (equal). **Yasuhiro Fujisawa:** Conceptualization (equal); data curation (equal); investigation (equal); methodology (equal); resources (equal); validation (equal); visualization (equal); writing – review and editing (equal). **Osamu Yamasaki:** Conceptualization (equal); data curation (equal); investigation (equal); methodology (equal); resources (equal); validation (equal); visualization (equal); writing – review and editing (equal). **Yasuhiro Nakamura:** Conceptualization (equal); data curation (equal); funding acquisition (equal); investigation (equal); methodology (equal); resources (equal); validation (equal); visualization (equal); writing – review and editing (equal). **Jun Asai:** Conceptualization (equal); data curation (equal); investigation (equal); methodology (equal); resources (equal); validation (equal); visualization (equal); writing – review and editing (equal). **Takeo Maekawa:** Conceptualization (equal); data curation (equal); investigation (equal); methodology (equal); resources (equal); validation (equal); visualization (equal); writing – review and editing (equal). **Takeru Funakoshi:** Conceptualization (equal); data curation (equal); investigation (equal); methodology (equal); resources (equal); validation (equal); visualization (equal); writing – review and editing (equal). **Shigeto Matsushita:** Conceptualization (equal); data curation (equal); investigation (equal); methodology (equal); resources (equal); validation (equal); visualization (equal); writing – review and editing (equal). **Eiji Nakano:** Conceptualization (equal); data curation (equal); investigation (equal); methodology (equal); resources (equal); validation (equal); visualization (equal); writing – review and editing (equal). **Kohei Oashi:** Conceptualization (equal); data curation (equal); funding acquisition (equal); investigation (equal); methodology (equal); resources (equal); validation (equal); visualization (equal); writing – review and editing (equal). **Junji Kato:** Conceptualization (equal); data curation (equal); investigation (equal); methodology (equal); resources (equal); validation (equal); visualization (equal); writing – review and editing (equal). **Hisashi Uhara:** Conceptualization (equal); data curation (equal); investigation (equal); methodology (equal); resources (equal); validation (equal); visualization (equal); writing – review and editing (equal). **Takuya Miyagawa:** Conceptualization (equal); data curation (equal); investigation (equal); methodology (equal); resources (equal); validation (equal); visualization (equal); writing – review and editing (equal). **Hiroshi Uchi:** Conceptualization (equal); data curation (equal); investigation (equal); methodology (equal); resources (equal); validation (equal); visualization (equal); writing – review and editing (equal). **Naohito Hatta:** Conceptualization (equal); data curation (equal); investigation (equal); methodology (equal); resources (equal); validation (equal); visualization (equal); writing – review and editing (equal). **Keita Tsutsui:** Conceptualization (equal); data curation (equal); investigation (equal); methodology (equal); resources (equal); validation (equal); visualization (equal); writing – review and editing (equal). **Taku Maeda:** Conceptualization (equal); data curation (equal); investigation (equal); methodology (equal); resources (equal); validation (equal); visualization (equal); writing – review and editing (equal). **Taisuke Matsuya:** Conceptualization (equal); investigation (equal); methodology (equal); resources (equal); validation (equal); visualization (equal); writing – review and editing (equal). **Hiroto Yanagisawa:** Conceptualization (equal); data curation (equal); investigation (equal); methodology (equal); resources (equal); validation (equal); visualization (equal); writing – review and editing (equal). **Ikko Muto:** Conceptualization (equal); data curation (equal); investigation (equal); methodology (equal); resources (equal); validation (equal); visualization (equal); writing – review and editing (equal). **Mao Okumura:** Conceptualization (equal); data curation (equal); investigation (equal); methodology (equal); resources (equal); validation (equal); visualization (equal); writing – review and editing (equal). **Dai Ogata:** Conceptualization (equal); data curation (equal); funding acquisition (equal); investigation (equal); methodology (equal); resources (equal); validation (equal); visualization (equal); writing – review and editing (equal). **Naoya Yamazaki:** Conceptualization (equal); data curation (equal); funding acquisition (lead); investigation (equal); methodology (equal); project administration (equal); resources (equal); supervision (equal); validation (equal); visualization (equal); writing – review and editing (equal).

## FUNDING INFORMATION

This work was supported in part by the National Cancer Center Research and Development Fund (2023‐J‐03).

## CONFLICT OF INTEREST STATEMENT

This research did not receive any specific grant or technical support from funding agencies in the commercial sectors or pharmaceutical companies. Outside the submitted work, Kenjiro Namikawa received honoraria from Ono pharmaceutical, Novartis, Bristol‐Myers Squibb, and MSD, and served as an advisory board for Novartis and MSD; Taiki Isei received honoraria from Ono pharmaceutical, Bristol‐Myers Squibb, and MSD; Tatsuya Takenouchi received honoraria from Ono pharmaceutical, Novartis, Bristol‐Myers Squibb, and MSD; Satoshi Fukushima received honoraria from Ono pharmaceutical, Novartis, and Bristol‐Myers Squibb; Takashi Inozume received honoraria from Ono pharmaceutical, Bristol‐Myers Squibb, and MSD; Yasuhiro Fujisawa received research grant from Ono pharmaceutical and Eisai, received honoraria from Ono pharmaceutical, Novartis, Bristol‐Myers Squibb, MSD, and Eisai, and served as an advisory board for Novartis and MSD; Osamu Yamasaki received research grant from Ono pharmaceutical, Daiichi‐Sankyo, and Maruho; Yasuhiro Nakamura received institutional research grant from Torii, received honoraria from Ono pharmaceutical, Novartis, Bristol‐Myers Squibb, MSD, Daiichi‐Sankyo, Maruho, Sun Pharma, and Tanabe Mitsubishi Pharma, and served as an advisory board for Novartis; Jun Asai received honoraria from Ono pharmaceutical and Novartis; Takeru Funakoshi received research grant from Ono pharmaceutical; Shigeto Matsushita received honoraria from Ono pharmaceutical, Novartis, Bristol‐Myers Squibb, and MSD; Hisashi Uhara received research grant from Ono pharmaceutical, and received honoraria from Ono pharmaceutical, Novartis, Bristol‐Myers Squibb, and MSD; Naoya Yamazaki received institutional research grant from Ono pharmaceutical, Novartis, Bristol‐Myers Squibb, Astellas Amgen BioPharma, Merck Serono, and Takara Bio, and received honoraria from Ono pharmaceutical, Novartis, Bristol‐Myers Squibb, and MSD, and served as an advisory board for Ono Pharmaceutical, Chugai Pharma, and MSD; other authors have no conflicts of interest to declare.

## ETHICS STATEMENT

Permission to perform the present study and a waiver for informed consent were obtained from the National Cancer Center Research Ethics Review Committee (IRB No. 2021‐238). Permission to perform the present study was obtained at each institution as well.

## Supporting information


Figure S1.

Figure S2.

Figure S3.

Table S1A–C.
Table S2.
Table S3.

## Data Availability

The data underlying this article will be shared on reasonable request to the corresponding author after obtaining additional permission from the National Cancer Center Research Ethics Review Committee.
