# Supplementary figures and images for: Systemic therapy for Asian patients with advanced BRAF V600‐mutant melanoma in a real‐world setting: A multi‐center retrospective study in Japan (B‐CHECK‐RWD study)
Source: Cancer Med. 2023 Aug 16;12(17):17967–80. doi: 10.1002/cam4.6438 (PMC10524053; doi:10.1002/cam4.6438)

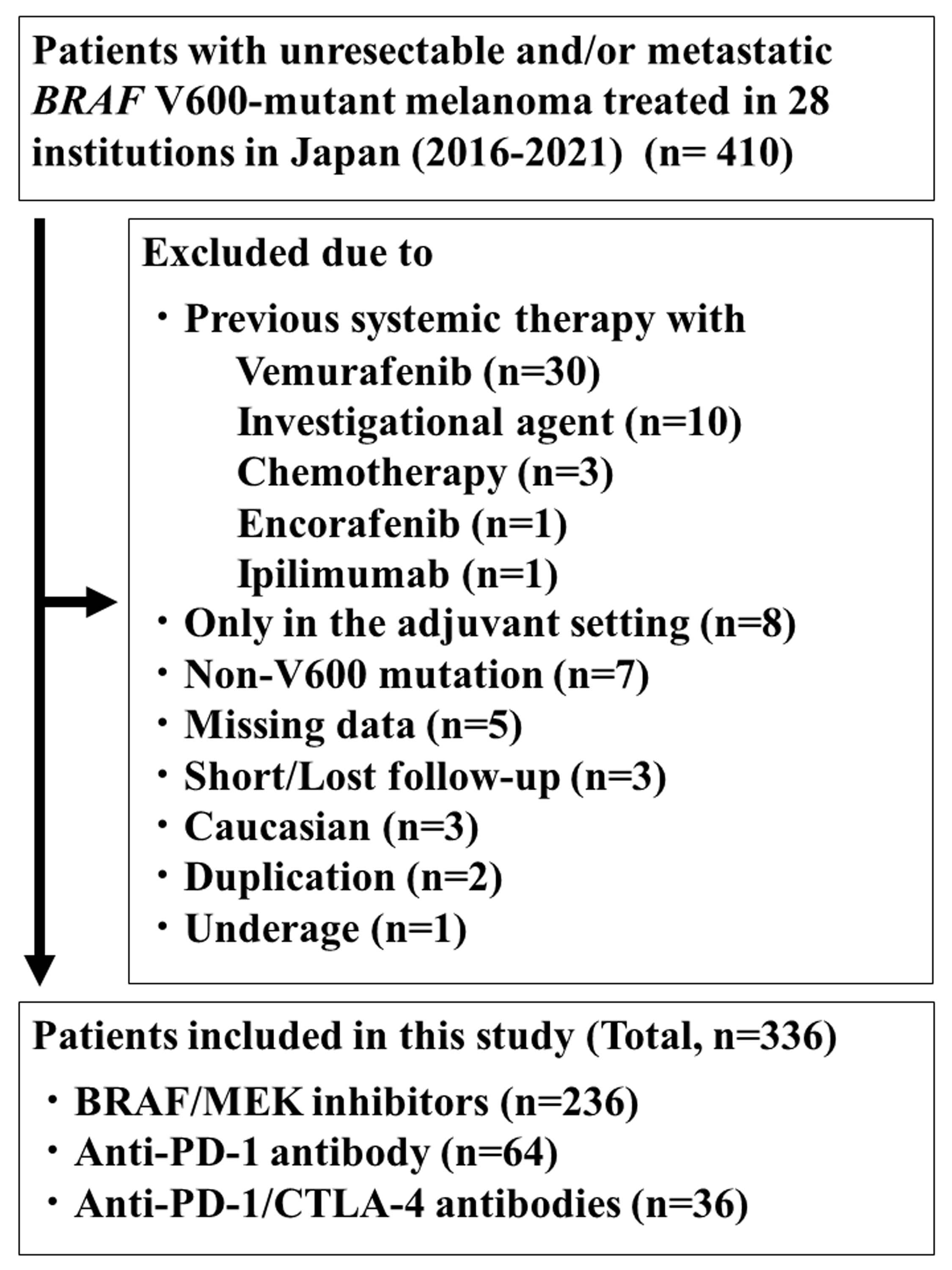

Supplement: Supplementary file 1 — Figure S1. [file CAM4-12-17967-s003.jpg]

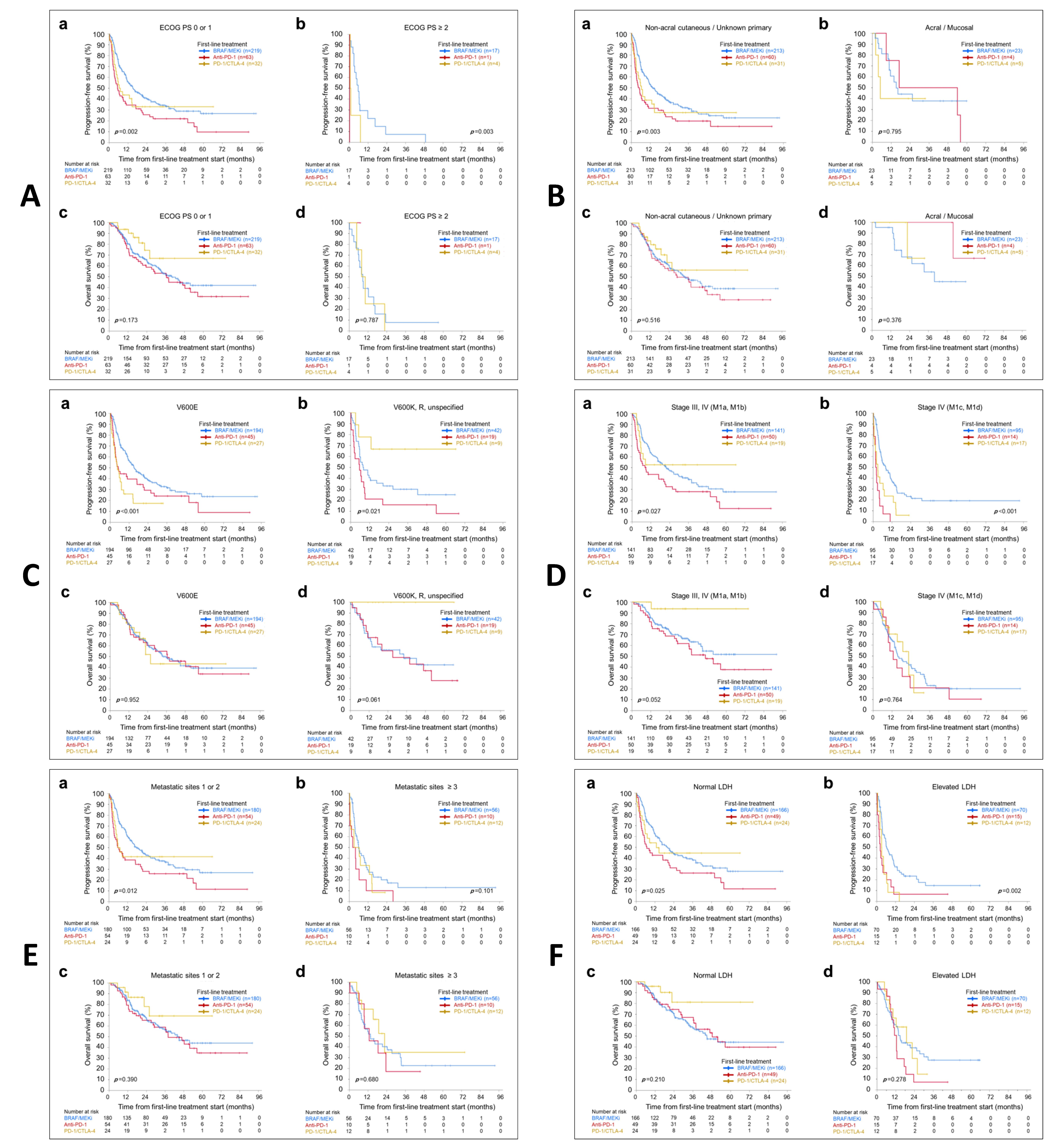

Supplement: Supplementary file 2 — Figure S2. [file CAM4-12-17967-s002.jpg]

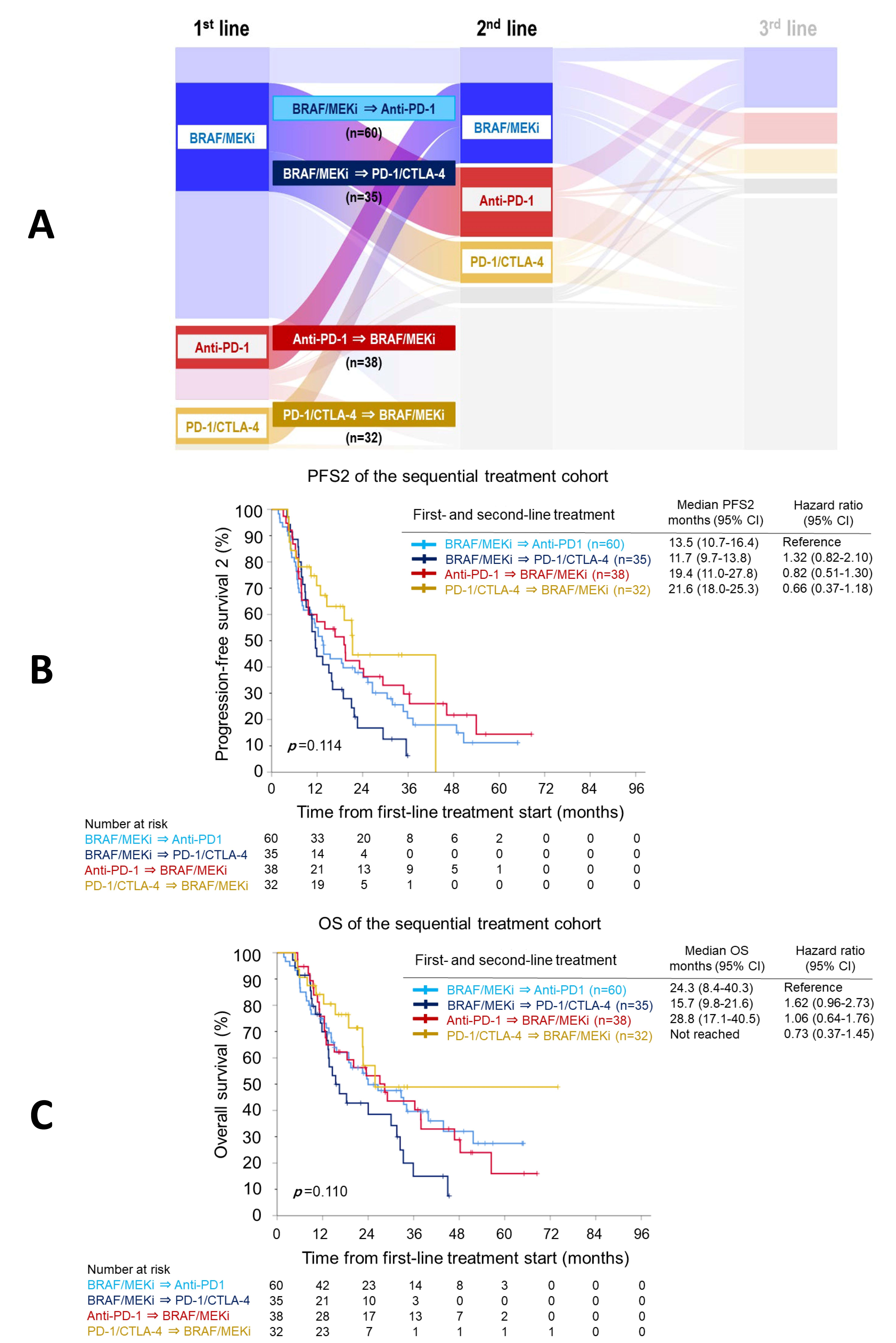

Supplement: Supplementary file 3 — Figure S3. [file CAM4-12-17967-s001.jpg]
