# Supplementary material for: Systemic therapy for Asian patients with advanced BRAF V600‐mutant melanoma in a real‐world setting: A multi‐center retrospective study in Japan (B‐CHECK‐RWD study)
Source: Cancer Med. 2023 Aug 16;12(17):17967–80. doi: 10.1002/cam4.6438 (PMC10524053; doi:10.1002/cam4.6438)
Supplement: Supplementary file 4 — Table S1A–C. Table S2. Table S3. [file CAM4-12-17967-s004.docx]

**Supplementary figure legends**

**Supplementary Figure S1**: Flow chart of the study population

**Supplementary Figure S2**: Kaplan–Meier curve for progression-free survival (PFS) (a, c) and overall survival (OS) (c, d) according to performance status (PS), PS 0-1 / ≥ 2 (A); melanoma subtype, non-acral cutaneous or unknown primary / acral or mucosal melanoma (B); BRAF genotype, BRAF V600E / V600K, R, or unspecified (C); AJCC stage, stage III or IV (M1a, M1b) / IV (M1c, M1d) (D); number of metastatic organ sites, 1-2 / ≥ 3 (E); and serum lactate dehydrogenase (LDH) at baseline, normal / elevated (F)

**Supplementary Figure S3**: Diagram of the treatment sequence and patient disposition from first- to second-line treatment (A) and Kaplan–Meier curve for progression-free survival 2 (PFS2) (B) and overall survival (OS) (C) according to sequential treatment pattern in patients who have received both first- and second-line treatment

Abbreviations: BRAF/MEKi, BRAF plus MEK inhibitors; Anti-PD-1, anti-PD-1 antibody; PD-1/CTLA-4, anti-PD-1 antibody plus anti-CTLA-4 antibody

**Supplementary Table S1A. Comparison of baseline characteristics of the patients receiving first-line BRAF/MEK inhibitors or anti-PD-1/CTLA-4 antibody in the original and matched cohort**

|  | Original cohort | |  | Matched cohort | |  |
| --- | --- | --- | --- | --- | --- | --- |
| Variables | BRAF/MEKi  (n = 236) | PD-1/CTLA-4  (n = 36) | SD | BRAF/MEKi  (n = 24) | PD-1/CTLA-4  (n = 24) | SD |
| Age |  |  |  |  |  |  |
| < 65 years | 132 (55.9) | 27 (75.0) | 0.410 | 18 (75.0) | 18 (75.0) | 0.000 |
| ≥ 65 years | 104 (44.1) | 9 (25.0) |  | 6 (25.0) | 6 (25.0) |  |
| Sex |  |  |  |  |  |  |
| Female | 119 (50.4) | 19 (52.8) | 0.048 | 12 (50.0) | 12 (50.0) | 0.000 |
| Male | 117 (49.6) | 17 (47.2) |  | 12 (50.0) | 12 (50.0) |  |
| ECOG PS |  |  |  |  |  |  |
| 0–1 | 219 (92.8) | 32 (88.9) | 0.136 | 22 (91.7) | 22 (91.7) | 0.000 |
| 2–4 | 17 (7.2) | 4 (11.1) |  | 2 (8.3) | 2 (8.3) |  |
| Subtype |  |  |  |  |  |  |
| NAC / UP | 213 (90.3) | 31 (86.1) | 0.130 | 22 (91.7) | 22 (91.7) | 0.000 |
| Acral / Mucosal | 23 (9.7) | 5 (13.9) |  | 2 (8.3) | 2 (8.3) |  |
| BRAF mutation |  |  |  |  |  |  |
| V600E | 194 (82.2) | 27 (75.0) | 0.176 | 19 (79.2) | 19 (79.2) | 0.000 |
| V600K / R / Unspecified | 42 (17.8) | 9 (25.0) |  | 5 (20.8) | 5 (20.8) |  |
| Stage (AJCC 8^th^ edition) |  |  |  |  |  |  |
| III / IV (M1a / M1b) | 141 (59.7) | 19 (52.8) | 0.139 | 16 (66.7) | 16 (66.7) | 0.000 |
| IV (M1c / M1d) | 95 (40.3) | 17 (47.2) |  | 8 (33.3) | 8 (33.3) |  |
| Metastatic organ sites |  |  |  |  |  |  |
| 1–2 | 180 (76.3) | 24 (66.7) | 0.214 | 18 (75.0) | 18 (75.0) | 0.000 |
| ≥ 3 | 56 (23.7) | 12 (33.3) |  | 6 (25.0) | 6 (25.0) |  |
| LDH |  |  |  |  |  |  |
| Normal | 166 (70.3) | 24 (66.7) | 0.078 | 17 (70.8) | 17 (70.8) | 0.000 |
| Elevated | 70 (29.7) | 12 (33.3) |  | 7 (29.2) | 7 (29.2) |  |

Abbreviations: BRAF/MEKi, BRAF plus MEK inhibitors; PD-1/CTLA-4, anti-PD-1 antibody plus anti-CTLA-4 antibody; ECOG PS, Eastern Cooperative Oncology Group performance status; AJCC, American Joint Committee on Cancer; LDH, lactate dehydrogenase; NAC, non-acral cutaneous; UP, unknown primary; SD, standardised difference

**Supplementary Table S1B. Comparison of baseline characteristics of the patients receiving first-line Anti-PD-1 or anti-PD-1/CTLA-4 antibody in the original and matched cohort**

|  | Original cohort | |  | Matched cohort | |  |
| --- | --- | --- | --- | --- | --- | --- |
| Variables | Anti-PD-1  (n = 64) | PD-1/CTLA-4  (n = 36) | SD | Anti-PD-1  (n = 19) | PD-1/CTLA-4  (n = 19) | SD |
| Age |  |  |  |  |  |  |
| < 65 years | 35 (54.7) | 27 (75.0) | 0.435 | 15 (78.9) | 15 (78.9) | 0.000 |
| ≥ 65 years | 29 (45.3) | 9 (25.0) |  | 4 (21.1) | 4 (21.1) |  |
| Sex |  |  |  |  |  |  |
| Female | 28 (43.8) | 19 (52.8) | 0.181 | 10 (52.6) | 10 (52.6) | 0.000 |
| Male | 36 (56.3) | 17 (47.2) |  | 9 (47.4) | 9 (47.4) |  |
| ECOG PS |  |  |  |  |  |  |
| 0–1 | 63 (98.4) | 32 (88.9) | 0.397 | 19 (100.0) | 19 (100.0) | 0.000 |
| 2–4 | 1 (1.6) | 4 (11.1) |  | 0 (0.0) | 0 (0.0) |  |
| Subtype |  |  |  |  |  |  |
| NAC / UP | 60 (93.8) | 31 (86.1) | 0.258 | 17 (89.5) | 17 (89.5) | 0.000 |
| Acral / Mucosal | 4 (6.3) | 5 (13.9) |  | 2 (10.5) | 2 (10.5) |  |
| BRAF mutation |  |  |  |  |  |  |
| V600E | 45 (70.3) | 27 (75.0) | 0.106 | 13 (68.4) | 13 (68.4) | 0.000 |
| V600K / R / Unspecified | 19 (29.7) | 9 (25.0) |  | 6 (31.6) | 6 (31.6) |  |
| Stage (AJCC 8^th^ edition) |  |  |  |  |  |  |
| III / IV (M1a / M1b) | 50 (78.1) | 19 (52.8) | 0.552 | 15 (78.9) | 15 (78.9) | 0.000 |
| IV (M1c / M1d) | 14 (21.9) | 17 (47.2) |  | 4 (21.1) | 4 (21.1) |  |
| Metastatic organ sites |  |  |  |  |  |  |
| 1–2 | 54 (84.4) | 24 (66.7) | 0.421 | 17 (89.5) | 17 (89.5) | 0.000 |
| ≥ 3 | 10 (15.6) | 12 (33.3) |  | 2 (10.5) | 2 (10.5) |  |
| LDH |  |  |  |  |  |  |
| Normal | 49 (76.6) | 24 (66.7) | 0.221 | 16 (84.2) | 16 (84.2) | 0.000 |
| Elevated | 15 (23.4) | 12 (33.3) |  | 3 (15.8) | 3 (15.8) |  |

Abbreviations: Anti-PD-1, anti-PD-1 antibody; PD-1/CTLA-4, anti-PD-1 antibody plus anti-CTLA-4 antibody; ECOG PS, Eastern Cooperative Oncology Group performance status; AJCC, American Joint Committee on Cancer; LDH, lactate dehydrogenase; NAC, non-acral cutaneous; UP, unknown primary; SD, standardised difference

**Supplementary Table S1C. Comparison of baseline characteristics of the patients receiving first-line BRAF/MEK inhibitors or Anti-PD-1 antibody in the original and matched cohort**

|  | Original cohort | |  | Matched cohort | |  |
| --- | --- | --- | --- | --- | --- | --- |
| Variables | BRAF/MEKi  (n = 236) | Anti-PD-1  (n = 64) | SD | BRAF/MEKi  (n = 59) | Anti-PD-1  (n = 59) | SD |
| Age |  |  |  |  |  |  |
| < 65 years | 132 (55.9) | 35 (54.7) | 0.024 | 31 (52.5) | 31 (52.5) | 0.000 |
| ≥ 65 years | 104 (44.1) | 29 (45.3) |  | 28 (47.5) | 28 (47.5) |  |
| Sex |  |  |  |  |  |  |
| Female | 119 (50.4) | 28 (43.8) | 0.133 | 26 (44.1) | 26 (44.1) | 0.000 |
| Male | 117 (49.6) | 36 (56.3) |  | 33 (55.9) | 33 (55.9) |  |
| ECOG PS |  |  |  |  |  |  |
| 0–1 | 219 (92.8) | 63 (98.4) | 0.276 | 59 (100.0) | 59 (100.0) | 0.000 |
| 2–4 | 17 (7.2) | 1 (1.6) |  | 0 (0.0) | 0 (0.0) |  |
| Subtype |  |  |  |  |  |  |
| NAC / UP | 213 (90.3) | 60 (93.8) | 0.130 | 57 (96.6) | 57 (96.6) | 0.000 |
| Acral / Mucosal | 23 (9.7) | 4 (6.3) |  | 2 (3.4) | 2 (3.4) |  |
| BRAF mutation |  |  |  |  |  |  |
| V600E | 194 (82.2) | 45 (70.3) | 0.282 | 44 (74.6) | 44 (74.6) | 0.000 |
| V600K / R / Unspecified | 42 (17.8) | 19 (29.7) |  | 15 (25.4) | 15 (25.4) |  |
| Stage (AJCC 8^th^ edition) |  |  |  |  |  |  |
| III / IV (M1a / M1b) | 141 (59.7) | 50 (78.1) | 0.406 | 47 (79.7) | 47 (79.7) | 0.000 |
| IV (M1c / M1d) | 95 (40.3) | 14 (21.9) |  | 12 (20.3) | 12 (20.3) |  |
| Metastatic organ sites |  |  |  |  |  |  |
| 1–2 | 180 (76.3) | 54 (84.4) | 0.205 | 51 (86.4) | 51 (86.4) | 0.000 |
| ≥ 3 | 56 (23.7) | 10 (15.6) |  | 8 (13.6) | 8 (13.6) |  |
| LDH |  |  |  |  |  |  |
| Normal | 166 (70.3) | 49 (76.6) | 0.143 | 47 (79.7) | 47 (79.7) | 0.000 |
| Elevated | 70 (29.7) | 15 (23.4) |  | 12 (20.3) | 12 (20.3) |  |

Abbreviations: BRAF/MEKi, BRAF plus MEK inhibitors; Anti-PD-1, anti-PD-1 antibody; ECOG PS, Eastern Cooperative Oncology Group performance status; AJCC, American Joint Committee on Cancer; LDH, lactate dehydrogenase; NAC, non-acral cutaneous; UP, unknown primary; SD, standardised difference

**Supplementary Table S2. Response to first-line, second-line, and third-line treatment**

|  | First-line treatment | | |  |
| --- | --- | --- | --- | --- |
|  | BRAF/MEKi  (n = 236) | Anti-PD-1  (n = 64) | PD-1/CTLA-4  (n = 36) | *p* value |
| Best overall response, n (%) |  |  |  |  |
| Complete response (CR) | 49 (20.8) | 13 (20.3) | 2 (5.6) |  |
| Partial response (PR) | 114 (48.3) | 4 (6.3) | 8 (22.2) |  |
| Stable disease (SD) | 36 (15.3) | 16 (25.0) | 8 (22.2) |  |
| Progressive disease | 22 (9.3) | 26 (40.6) | 13 (36.1) |  |
| Not evaluable | 15 (6.4) | 5 (7.8) | 5 (13.9) |  |
| Objective response rate  n (%), [95% CI] | 163 (69.1),  [62.7–74.9] | 17 (26.6),  [16.3–39.1] | 10 (27.8),  [14.2–45.2] | < 0.001 |
| Disease control rate  n (%), [95% CI] | 199 (84.3),  [79.0–88.7] | 33 (51.6),  [38.7–64.2] | 18 (50.0),  [32.9–67.1] | < 0.001 |
|  | Second-line treatment | | |  |
|  | BRAF/MEKi  (n = 101) | Anti-PD-1  (n = 61) | PD-1/CTLA-4  (n = 36) | *p* value |
| Best overall response, n (%) |  |  |  |  |
| Complete response (CR) | 15 (14.9) | 6 (9.8) | 1 (2.8) |  |
| Partial response (PR) | 50 (49.5) | 3 (4.9) | 5 (13.9) |  |
| Stable disease (SD) | 21 (20.8) | 15 (24.6) | 5 (13.9) |  |
| Progressive disease | 6 (5.9) | 33 (54.1) | 21 (58.3) |  |
| Not evaluable | 9 (8.9) | 4 (6.6) | 4 (11.1) |  |
| Objective response rate  n (%), [95% CI] | 65 (64.4),  [54.2–73.6] | 9 (14.8),  [7.0–26.2] | 6 (16.7),  [6.4–32.8] | < 0.001 |
| Disease control rate  n (%), [95% CI] | 86 (85.1),  [76.7–91.4] | 24 (39.3),  [27.1–52.7] | 11 (30.6),  [16.3–48.1] | < 0.001 |
|  | Third-line treatment | | |  |
|  | BRAF/MEKi  (n = 54) | Anti-PD-1  (n = 27) | PD-1/CTLA-4  (n = 21) | *p* value |
| Best overall response, n (%) |  |  |  |  |
| Complete response (CR) | 3 (5.6) | 2 (7.4) | 1 (4.8) |  |
| Partial response (PR) | 14 (25.9) | 2 (7.4) | 4 (19.0) |  |
| Stable disease (SD) | 17 (31.5) | 7 (25.9) | 4 (19.0) |  |
| Progressive disease | 11 (20.4) | 12 (44.4) | 9 (42.9) |  |
| Not evaluable | 9 (16.7) | 4 (14.8) | 3 (14.3) |  |
| Objective response rate  n (%), [95% CI] | 17 (31.5),  [19.5–45.6] | 4 (14.8),  [4.2–33.7] | 5 (23.8),  [8.2–47.2] | 0.251 |
| Disease control rate  n (%), [95% CI] | 34 (63.0),  [48.7–75.7] | 11 (40.7),  [22.4–61.2] | 9 (42.9),  [21.8–66.0] | 0.100 |

Abbreviations: BRAF/MEKi, BRAF plus MEK inhibitors; Anti-PD-1, anti-PD-1 antibody, PD-1/CTLA-4, anti-PD-1 antibody plus anti-CTLA-4 antibody; CI, confidence interval; the exact method based on binomial distributions (Clopper–Pearson 95% CIs) is used to determine response and disease control rates

**Supplementary Table S3. Grade 3 or higher adverse events and any grade adverse events leading to treatment discontinuation**

|  | First-line treatment | | |
| --- | --- | --- | --- |
| Adverse events, n (%) | BRAF/MEKi  (n = 236) | Anti-PD-1  (n = 64) | PD-1/CTLA-4  (n = 36) |
| Pyrexia | 32 (13.6) | 0 (0.0) | 2 (5.6) |
| AST/ALT increased | 13 (5.5) | 1 (1.6) | 7 (19.4) |
| CPK increased | 15 (6.4) | 0 (0.0) | 0 (0.0) |
| Nausea / Vomiting | 7 (3.0) | 0 (0.0) | 0 (0.0) |
| Diarrhoea / Colitis | 1 (0.4) | 1 (1.6) | 5 (13.9) |
| Uveitis | 4 (1.7) | 0 (0.0) | 2 (5.6) |
| Neutrophil count decreased | 5 (2.1) | 0 (0.0) | 0 (0.0) |
| Rash | 4 (1.7) | 1 (1.6) | 0 (0.0) |
| Pneumonitis | 1 (0.4) | 3 (4.7) | 0 (0.0) |
| Fatigue | 3 (1.3) | 0 (0.0) | 0 (0.0) |
| Anemia | 3 (1.3) | 0 (0.0) | 0 (0.0) |
| Serous retinal detachment | 3 (1.3) | 0 (0.0) | 0 (0.0) |
| Hypophysitis | 0 (0.0) | 2 (3.1) | 1 (2.8) |
| Adrenal insufficiency | 0 (0.0) | 1 (1.6) | 2 (5.6) |
| Type 1 diabetes / hyperglycaemia | 0 (0.0) | 1 (1.6) | 2 (5.6) |
| Hyper- / Hypothyroidism | 0 (0.0) | 0 (0.0) | 3 (8.3) |
| Blood bilirubin increased | 2 (0.8) | 0 (0.0) | 0 (0.0) |
| Platelet count decreased | 2 (0.8) | 0 (0.0) | 0 (0.0) |
| Deep vein thrombosis | 2 (0.8) | 0 (0.0) | 0 (0.0) |
| Arthralgia | 2 (0.8) | 0 (0.0) | 0 (0.0) |
| Myalgia | 1 (0.4) | 0 (0.0) | 1 (2.8) |
| Nephritis | 1 (0.4) | 0 (0.0) | 1 (2.8) |
| Gastritis | 0 (0.0) | 1 (1.6) | 1 (2.8) |
| Anorexia | 1 (0.4) | 0 (0.0) | 0 (0.0) |
| Eye pain | 1 (0.4) | 0 (0.0) | 0 (0.0) |
| Arrhythmia | 1 (0.4) | 0 (0.0) | 0 (0.0) |
| Sudden death | 1 (0.4) | 0 (0.0) | 0 (0.0) |
| Lipase increased | 0 (0.0) | 1 (1.6) | 0 (0.0) |
| Infusion reaction | 0 (0.0) | 1 (1.6) | 0 (0.0) |
| Skin infection | 0 (0.0) | 1 (1.6) | 0 (0.0) |
| Conjunctivitis infective | 0 (0.0) | 1 (1.6) | 0 (0.0) |
| Encephalitis | 0 (0.0) | 1 (1.6) | 0 (0.0) |
| Hypertension | 0 (0.0) | 0 (0.0) | 1 (2.8) |
| Ejection fraction decreased | 0 (0.0) | 0 (0.0) | 1 (2.8) |
| Haemophagocytic syndrome | 0 (0.0) | 0 (0.0) | 1 (2.8) |
| Total* | 82 (34.7) | 14 (21.9) | 27 (75.0) |

Abbreviations: BRAF/MEKi, BRAF plus MEK inhibitors; Anti-PD-1, anti-PD-1 antibody; PD-1/CTLA-4, anti-PD-1 antibody plus anti-CTLA-4 antibody; AST, aspartate aminotransferase; ALT, alanine aminotransferase; CPK, creatinine phosphokinase

* Number of patients with either grade 3 or higher adverse event or any grade of adverse event leading to treatment discontinuation
